# Exciton diffusion exceeding 1 µm: run, exciton, run!

**DOI:** 10.1038/s41377-021-00480-2

**Published:** 2021-02-22

**Authors:** Ibrahim Dursun, Burak Guzelturk

**Affiliations:** 1 Department of Electrical Engineering, The Pennsylvania State University, University Park, PA 16802 USA; 2 X-ray Science Division, Advanced Photon Source, Argonne National Laboratory, Lemont, IL USA

**Keywords:** Physics, Optical physics

## Abstract

Exciton diffusion lengths reaching the micrometer length scale have long been desired in solution-processed semiconductors but have remained unattainable using conventional materials to date. Now halide perovskite nanocrystal films show unprecedented exciton migration with diffusion lengths approaching 1 µm owing to the efficient combination of radiative and nonradiative energy transfer.

Excitons, bound electron-hole pairs, govern the optoelectronic functionalities of quantum-confined and molecular semiconductors, which are commonly used in photovoltaic devices and artificial photosynthesis. In such light-harvesting applications, it is desired to achieve long exciton diffusion lengths to maximize exciton dissociation at interfaces to boost energy conversion efficiencies. Nevertheless, excitonic devices have long suffered from limited exciton diffusion lengths. In typical solution-processed materials such as conjugated polymers and colloidal quantum dots, exciton diffusion is limited to a length scale on the order of 10 nm due to energetic disorder in thin films and weak energy transfer interactions between chromophores^1^.

Researchers have long pursued materials that can sustain long exciton diffusion lengths. In recent years, these efforts have identified new materials^2–4^ with exciton diffusion lengths larger than 100 nm (Fig. 1a) enabled by minimized nanoscale energetic disorder and enhanced energy transfer interactions. Surpassing the diffusion length barrier of 100 nm has been critical for light-harvesting applications to match the light absorber layer thickness to the diffusion length. Therefore, long-range exciton diffusion (Fig. 1b) is expected to boost the efficiencies of excitonic devices, yet ultralong exciton diffusion lengths approaching 1 µm have been considered inconceivable to date.

**Fig. 1:**
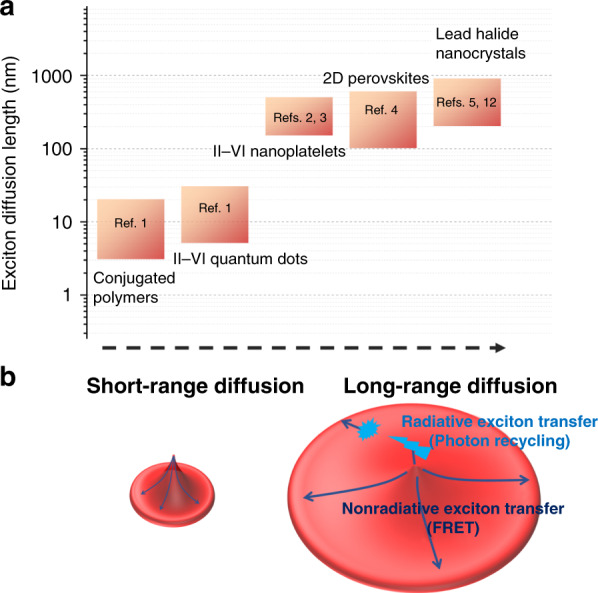
Exciton diffusion in soft semiconductors. **a** Exciton diffusion length in various solution-processed semiconductors. **b** Schematic for short-range exciton diffusion in a system with high energetic disorder and weak dipole–dipole coupling. Long-range exciton diffusion in a system with strong dipole–dipole coupling and radiative energy transfer

A report by Giovanni and colleagues now shows that thin films of methylammonium lead bromide (MAPbBr_3_) perovskite nanocrystals (PNCs) achieve unprecedented long-range exciton diffusion surpassing the 1 μm barrier, which was measured and quantified by a steady-state photoluminescence imaging method^5^. Radiative and nonradiative energy transfer pathways have been considered to underpin the long-range diffusion found in PNC thin films. They found that radiative energy transfer via photon recycling^6–8^ assists exciton diffusion, while the dominant contribution comes from nonradiative energy transfer among PNCs via near-field dipole–dipole coupling, a mechanism known as Förster resonance energy transfer (FRET).

To promote exceptional exciton diffusion, PNCs bring together several favorable photophysical properties that include high defect tolerance, high photoluminescence quantum yields, and large intrinsic absorption cross-sections^9,10^, by which exciton hopping via FRET among PNCs is maximized while undesired exciton trapping is minimized. In addition, a small Stokes shift and small inhomogeneous broadening^11^ enhance radiative energy transfer among PNCs through photon recycling. Giovanni et al. quantified the exciton mobility in PNC films through measuring exciton diffusion coefficients^5^, which were found to be 10 cm^2^ V^−1^ s^−1^, on par with the charge carrier mobilities commonly found in polycrystalline lead halide thin films. The exceptionally large diffusivity of excitons shows the strong promise of PNCs as active materials in solution-processed optoelectronics rivaling their bulk halide perovskite counterparts.

Another recent study^12^ examined exciton diffusion in an all-inorganic PNC of cesium lead bromide (CsPbBr_3_), which showed diffusion lengths exceeding 200 nm. Achievement of long-range diffusion in both MAPbBr_3_ and CsPbBr_3_ PNCs indicates that strong exciton migration is a common trait of the PNC family. Longer exciton diffusion lengths reported in PNCs with methylammonium cations^5^ may hint at a better defect tolerance associated with organic cations. Nevertheless, the effects of A-site cations and PNC composition on exciton diffusion properties remain to be understood by future studies. Furthermore, Giovanni et al. showed that organic ligand molecules surrounding PNCs have a role in determining the extent of exciton diffusion. PNCs with octylamine ligands are found to have the longest exciton diffusion length compared to those surrounded by oleylamine and hexylamine ligands, indicating that surface chemistry is another feature that can be used to control exciton transport. Overall, further work is needed to decipher the effects of chemical composition and surface chemistry in addition to the nanoscale orientation and stacking of PNCs in their thin films.

In determining the strength of nonradiative energy transfer, the Förster radius is an important figure of merit; this radius is the distance between two energy transferring species when the energy transfer efficiency is 50%. In MAPbBr_3_ PNCs, the Förster radius is estimated to be ca. 13 nm based on the linear absorption cross-section, photoluminescence quantum yield, and spectral overlap between emission and absorption in the PNCs. Under the isotropic exciton diffusion assumption, one can estimate the exciton diffusion length via the Einstein relation:^5^ $L_D = AR_0^3/d^2$, where *L*_*D*_ is the diffusion length, *R*_0_ is the Förster radius, d is the dipole–dipole separation and A is a constant related to the distance distribution between particles (A is typically close to 1). Using the estimated Förster radius, one can estimate an exciton diffusion length of ca. 60 nm. However, the experimental exciton diffusion length is found to be an order of magnitude larger, which would translate into a Förster radius larger than 30 nm. This deviation in the strength of nonradiative energy transfer implies that either exciton diffusion in PNC films is highly anisotropic or exciton hopping between adjacent PNCs goes beyond simple dipole-dipole coupling theory due to the strong oscillator strength of the PNCs. In addition, collective coherent coupling among PNCs may result in superdiffusive (i.e., ballistic) exciton migration, yet a comprehensive understanding of the microscopic mechanisms behind efficient exciton transport in PNCs would require future studies involving spatiotemporal tracking of exciton motion.

After decades of research, emerging solution-processed materials are now attaining ultralong range exciton diffusion. Giovanni et al. unlocked the micron-scale exciton diffusion regime by using halide perovskite-based nanocrystals and provided an important piece of the puzzle towards efficient excitonic devices and light-harvesting systems. Importantly, key attributes of PNCs, including high defect tolerances and strong light-matter interactions (i.e., large oscillator strength and absorption cross-section) enabling long-range exciton diffusion, will provide blueprints for next-generation excitonic materials towards superior excitonic transport that can enable devices such as excitonic transistors.
